# Effects of hormonal contraceptives on lipid profile among women attending family planning unit in Goba Town Public Health Facilities, Bale, Southeast Ethiopia: a comparative cross-sectional study

**DOI:** 10.1186/s12978-023-01727-4

**Published:** 2023-12-19

**Authors:** Awel Turki, Almaz Ayalew, Andualem Mossie, Shimelis Mitiku

**Affiliations:** 1 Department of Physiology, Goba Referral Hospital, Madda Walabu University, Bale, Ethiopia; 2 Department of Physiology, Saint Paul’s Millenium Medical College, Addis Ababa, Ethiopia; 3 Department of Biomedical Sciences, Institute of Health, Jimma University, Jimma, Ethiopia

**Keywords:** Hormonal contraceptives, Lipid profile, Depo-Medroxyprogesterone Acetate, Implant, Progesterone only pills, Southeast Ethiopia

## Abstract

**Background:**

Hormonal contraceptives are a widely used contraceptive method for the prevention of pregnancy in women. It is associated with change in lipid profile which results in congestive heart failure, coronary heart disease, angina, deep vein thrombosis and stroke which are the major cause of premature death. We aim to investigate the effects of hormonal contraceptive use on lipid profile among women attending family planning unit in Goba Town Public Health Facilities.

**Methods:**

A comparative cross-sectional study design was conducted on 93 hormonal contraceptive users and 93 non-users’ women in Goba Town Public Health Facilities from September to November, 2022. Blood samples for the estimation of TC, TG, HDL-c and LDL-c levels were collected. Student's independent t-test was used to compare the results of lipid profiles. One-way ANOVA was used to identify the variation of lipid profile between progestin only pills, DMPA and implant users. Simple linear regression was used to determine the change in lipid profiles in relation to the duration of hormonal contraceptive use. P-value less than 0.05 was considered as statistically significant.

**Result:**

The mean serum level of TC, TG and LDL-c was significantly increases in hormonal contraceptive users in comparison with non-users. The mean serum level TC, TG, LDL-c and HDL-c was significantly different between DMPA, implanon and POP users. The mean serum level of TC, TG and LDL-c in implanon users was lower than DMPA and POP users. As the duration of DMPA and POP use increases, the serum level of TC, TG and LDL-c were significantly increased. But, the serum level of HDL-c was significantly decreased. LDL-c was significantly increased with the duration of implanon use.

**Conclusion:**

The mean serum level of TC, TG and LDL-c were significantly increased among hormonal contraceptive users. The mean serum level of lipid profile was significantly different between DMPA, implanon and POP users. The serum level of TC, TG, LDL-c were directly proportional to the duration of DMPA and POP use. Routine evaluation of lipid profiles is advisable before and after initiation of hormonal contraceptives.

## Background

Family planning refers to the use of various methods of fertility control that will help individuals (men and women) or couples to have the number of children they want and when they want them in order to assure the wellbeing of children and the parents [1, 2]. Hormonal contraceptives are widely used for the prevention of pregnancy in women. They are available in many forms including oral combined pills which are made of a combination of estrogen and progesterone, progesterone only pills, Depo-Medroxyprogesterone Acetate (DMPA) injection and implant [3].

Long term use of hormonal contraceptives was associated with different metabolic effects including change in carbohydrate metabolism, body weight, blood pressure, liver enzymes, and lipid profile which result in coronary heart disease, congestive heart failure, angina, peripheral arterial disease, deep vein thrombosis, and stroke which are the major cause of premature death [4, 5].

Different methods of hormonal contraceptives; progesterone only contraceptives, DMPA and implant is the most popular contraceptive method in Sub-Saharan Africa including Ethiopia, contributing 48% of total method mix in the region [6]. In Ethiopia, the contraceptive prevalence rate is 36% for all women and 42% for currently married women. The vast majority of women use modern methods than traditional methods. Among currently married women, the most popular methods are injectable (23%), implants (8%), IUCD, and the pill (2%) [7].

Various synthetic progestin that are used as contraceptives have been reported to influence lipid profile [8]. Lipid profile abnormalities as a result of hormonal contraceptives use was associated with some adverse effects including increase in serum glucose, body weight, blood pressure and atherosclerosis [9]. It is now generally accepted that elevated levels of non-high density lipoprotein-c (HDL-c) and low HDL-c concentrations may promote the development of atherosclerosis that may influence the development of cardiovascular disease (CVD) [10].

Study revealed that, one-third of ischemic heart diseases in the world are secondary to hypercholesterolemia, and it is estimated that hypercholesterolemia is responsible for 2.6 million (4.5%) deaths in the world [11]. The burden of CVD secondary to dyslipidemia is a challenging task in sub-Saharan African countries including Ethiopia [12]. In 2017, the World Health Organization (WHO) reported that around 9% of the Ethiopian population died due to CHD secondary to lipid profile abnormalities [13]. A systematic review conducted in Ethiopia found that from 9% of Coronary Heart Disease death due to dyslipidemia, 5.2% were women of reproductive age those use hormonal contraceptives [14]. The resultant effect of contraceptive hormones on lipid metabolism depends on the type and duration of treatment [15].

A number of research investigations have been conducted on the relationship between hormonal contraceptives and lipid profiles, results are not consistent between studies. For example, some have demonstrated that hormonal contraceptives do not affect serum lipids [16–18]. While others have shown an adverse relationship [19, 20] and others reported beneficial effect [12, 21]. In general, the result of study conducted on the relationship between hormonal contraceptives and lipid profiles are also not consistent.

There is also an information gap on the exact effect of hormonal contraceptives on lipid profiles among Ethiopian women and this information is critical to ensure optimum delivery of comprehensive follow-up service. The present study was aim to assess the effect of hormonal contraceptives on lipid profile and provide information for those users of hormonal contraceptives as well as for clinician and family planning service care providers for early detection of lipid profile before it results in serious complication and death. It also provides baseline data for further investigations to researchers and policy makers who want to do more on the effect of hormonal contraceptives on lipid profile.

## Methods

### Study area and period

This study was conducted in all Goba Town Public Health Facilities (Madda Walabu University Goba Referral Hospital, Goba Health Center and Harawa Sinja Health Center) from September to November, 2022. The study area-Goba town is located in the Bale Zone, Oromia National Regional State, Southeast Ethiopia. Goba town is located at a distance of 430 km away from the capital of Ethiopia- Addis Ababa and 12 km from Robe town.

### Study design and population

Facility based multi-center comparative cross-sectional study design was conducted among women attending family planning unit in Goba Town Public Health Facilities. The study population were comprised of women between 15 and 49 years of age, those users of hormonal contraceptives, attending the family planning unit in Goba Town Public Health Facilities for follow up or discontinuation of service, apparently healthy women, who voluntarily consent to participate in the study, fulfill inclusion criteria and selected as a sample (for users). The other study population were comprised non-users of hormonal contraceptives methods attending the family planning unit in Goba Town Public Health Facilities for follow up or discontinuation of service in those users of IUCD or new comers plan to seek any type of contraception, apparently healthy women, who voluntarily consent to participate in the study, fulfill inclusion criteria and selected as a sample of the same age and BMI with users (for non-users).

For hormonal contraceptives users, women between 15 and 49 years of ages who have been using progestin only pills, DMPA and implanon for at least 3 months; and for non-users, women between 15 and 49 years of ages, women who have been using IUCD and new visitors those plan to use any type of contraception were included in the study.

Women with known chronic disease (diabetes, hypertension, liver dysfunction, cardiovascular disorders); pregnant women; women with chronic alcohol and/or tobacco use; BMI ≥ 30 or obesity; and those currently using medication known to affect lipid profile (including corticosteroids, antipsychotics, diuretics, anticonvulsants, retinoid) were excluded by reviewing their medical records and/or by asking. For non-user, women who have been using any hormonal contraceptives during the last 1 year were also excluded.

### Sample size determination and sampling technique

The sample size was calculated by using mean estimating formula. The value of the outcome variable was taken from the previous study done in Ethiopia [20]. After calculating all the values of lipid profile components, the component with the highest value (total cholesterol) was taken. The reported mean ± SD values of total cholesterol for users were (183.12 ± 40.56) whereas, (161.76 ± 29.45) for non-users. By assuming; significance level = 95%, 80% power of the test, type of test = two-sided, Z α\2 = the critical value at 95% confidence level of certainty (1.96).

Therefore, the value of sample size calculated was 56. By considering design effect of 1.5 (56*1.5 = 84) because the samples were taken from different institution, the calculated sample size was 84. Since equal number of cases and controls was used, nˣ2 = 84ˣ2 = 168. Taking non-response rate of 10% = 10/100ˣ168 = 18. Then the total sample size was 186 (168 + 18). So in our study, 93 hormonal contraceptive users who fulfill inclusive criteria of case and 93 non-users that fulfill inclusive criteria of control were included.

First, the three public health facilities (MWU GRH, Goba Health Center and Harawa Sinja Health Center) were included. Sample size was proportionally allocated for each health facilities using proportional allocation formula. Then, a systematic random sampling technique was used to select study participants in each health facilities. i.e. every 2nd values was selected according to their order of visiting by using K = N/n formula.

### Operational definitions

Castelli Risk Index: are lipid ratios estimated as TC/HDL-c and LDL-c/HDL-c to predict cardiovascular disease risk.

Castelli Risk Index Classification: If the difference in the ratio of TC/HDL-c (Castelli Risk Index I) or LDL-c/HDL-c (Castelli Risk Index II) between hormonal contraceptive users and non-users are < 0.11 (low risk), 0.11–0.21 (intermediate risk), > 0.21 (high risk) for development of CVD.

Contraceptives: birth control methods that include pills, Depo-Provera, IUCD, implant, natural or surgical procedures to prevent conception.

Coronary Heart Disease: chest pain or heart attack due to atherosclerosis which is secondary to excess level of TC, LDL-c and TG.

Hormonal Contraceptive users: Women who have been using progestin only pills, Depo-Provera and implanon.

Lipid Profile: a panel of blood test of TC, TG, HDL-c and LDL-c.

Non-hormonal Contraceptive users: Women who have been using IUCD, natural methods and surgical method.

### Data collection tools and procedures

Socio-demographic characteristics, type and duration of contraceptive use was collected from the participants by using Afan Oromo and Amharic version of the semi-structured questionnaire by trained midwifery professional. Five milliliter of venous blood was collected using sterile syringes and needles from each participant using aseptic procedure by laboratory technologist. Then, the blood samples were left for 30 to 40 min at Room Temperature, centrifuged at 3000 RPM for 5 min and serum of blood samples were separated. Estimation of lipid profiles (TC, TG, LDL-c and HDL-c), were conducted using Bio-system mindray BS-200 Chemistry Analyzer. TC/HDL-c ratio (Castelli index I) and LDL-c/HDL-c (Castelli index II) were calculated to determine the CVD risk. Weight and height was measured by using weight scale and stadiometer respectively. BMI was calculated from weight in kg/ height in m^2^.

### Data entry and statistical analysis

Data entry was done using Epidata software version 4.6.0.2. SPSS software (version 25; for windows) was used for data analysis. After complete entry of all the data, soft copy was checked with its hard copy to see the consistency. Standard statistical methods are used to determine the mean, standard deviation (SD) and the range. Student's independent t-test was used to compare the results of lipid profiles of hormonal contraceptive users and control group. One-way ANOVA (analysis of variance) was used to identify the variation of lipid profile between progestin only pills, DMPA and implant users. Simple linear regression was used to determine the change in lipid profile in relation to the duration of hormonal contraceptive use. The normality and homoscedasticity of the data were checked by using the kolmogrov-smirnov test and Levene’s test respectively. Variables with P-value < 0.05 was considered as a statistically significant. Finally, the result was present using graph, table and text.

### Data quality control

Training on the contents of the questionnaire, data collection techniques, and research ethics was given for data collectors. The quality of the data was controlled carefully. Close supervision was done by the principal investigator throughout the data collection. Collected data checked for completeness and consistency daily. The completed questionnaires were rechecked repeatedly by the principal investigator to maintain the quality of data. Pretest of the questionnaire was conducted in 5% study participant at Robe General Hospital for 1 week prior to actual data collection to check tools reliability.

### Ethical consideration

Ethical clearance was obtained from Jimma University, Institutional Review Board (Ref. No: 73/22); a support letter was obtained from the Health Research and Post Graduate Director’s Office for each study area and concerned health facilities was communicated with formal letter (Ref. No: H/I/R/P/G/D/63/2022). Written consent was obtained from each study participant. Each study participant was informed about the research, and confidentiality of information was maintained during data collection, analysis, interpretation and publication of results. Findings were communicated in aggregated form and individual information was kept confidentially. The culture, religion and society values were highly respected in every concern during data collection. COVID-19 prevention protocols were taken for both data collectors and respondents during data collection. Clients with elevated lipid profiles were linked for further investigation and treatment.

## Results

### Socio-demographic characteristics of the study participants

A total of 186 study participants (93 hormonal contraceptive users and 93 non-users (controls) women) were included. Among hormonal contraceptive users, 37 were DMPA users, 32 were implant users and 24 were progesterone only pills users. The mean age (years) of the hormonal contraceptives users were 29.31 ± 4.67; while, control groups were 28.08 ± 4.40 which were not significantly different (p = 0.72). The age ranges in hormonal contraceptive users were 19–39 years and 20–37 years in controls. Majority of study participants, 54 (58.1%) of hormonal contraceptive users and 56 (59.1%) of the non-users were aged 20–30 years, 3 (3.2%) of HC users and 2 (2.7%) non-users were women aged less than 20 years in both groups. Whereas, 36 (38.7%) of hormonal contraceptive users and 35 (38.2%) of the controls were aged greater than 30 years.

More than half of hormonal contraceptive users 63 (67.7%) and 71 (76.3%) non-users were Oromo whereas 45 (48.4%) of users and 49 (52.7) of non-users were Muslim. Eighty-four (90.3%) of hormonal contraceptive users and 82 (88.2%) of non-users were married; while 73 (78.5%) of hormonal contraceptive users and 70 (75.3%) of non-users had secondary and above secondary school educational status. Sixty-one (65.6%) of hormonal contraceptive users and 55 (59.1%) of non-users were live in urban area.

The mean Body Mass index (kg/m^2^) of the two groups were 21.67 ± 1.94 (hormonal contraceptive users) and 21.65 ± 1.93 (non-users) and was not significantly different (p = 0.93). Majority, 89.3% of hormonal contraceptive users and 82 (88.2%) of non-contraceptive users had a BMI of between 18.5 and 24.9 kg/m^2^ as presented in Table 1.

**Table 1:** Socio-demographic characteristics of the study participants in Goba Town Public Health Facilities, Bale, Southeast Ethiopia, 2022

Variables	Hormonal Contraceptives		P-value
	User n (%) (n = 93	Non-User n (%) (n = 93)	
Age (in years)			
< 20	3 (3.2)	2 (2.7)	
20–30	54 (58.1)	56 (59.1)	0.88
> 30	36 (38.7)	35 (38.2)	
Ethnicity			
Oromo	63 (67.7)	71 (76.3)	
Amhara	28 (30.1)	20 (21.5)	0.4
Sidama	2 (2.2)	2 (2.5)	
Religion			
Muslim	45 (48.4)	49 (52.7)	
Orthodox	36 (38.7)	23 (24.7)	0.06
Protestant	12 (12.9)	21 (22.6)	
Marital status			
Single	2 (2.2)	2 (2.2)	
Married	84 (90.3)	82 (88.2)	0.87
Divorced	7 (7.5)	9 (9.7)	
Educational level			
Illiterate	3 (3.2)	1 (1.1)	
Primary	17 (18.3)	22 (23.7)	0.61
Secondary	32 (34.4)	29 (31.2)	
Above secondary	41 (44.1)	41 (44.1)	
Occupation			
Private	69 (74.2)	74 (79.6)	0.38
Government	24 (25.8)	19 (20.4)	
Monthly Income (ETB*)			
< 1400	1 (1.1)	1 (1.1)	
1400–3500	54 (58.1)	54 (58.1)	
3501–5000	24 (21.5)	33 (35.5)	0.18
> 50,000	18 (19.4)	5 (5.4)	
Residence			
Urban	61 (65.6)	55 (59.1)	0.36
Rural	32 (34.4)	38 (40.9)	
BMI**			
< 18.5 (Underweight)	3 (3.2)	2 (2.2)	
18.5–24.9 (Normal)	83 (89.2)	82 (88.2)	0.79
> 25 (Overweight)	7 (7.5)	9 (9.7)	

*Ethiopian Birr, **Body Mass Index

### Lipid profile status of the study participants

The mean serum TC levels in hormonal contraceptive users were 151.00 ± 22.67 mg/dl which was significantly higher (P = 0.000) by 13.52 mg/dl than that of non-users (137.48 ± 22.65 mg/dl). The maximum serum TC level among hormonal contraceptive users and non-users were 196.7 mg/dl; and 180.2 mg/dl whereas 102.6 mg/dl and 100.2 mg/dl were the minimum serum TC level among hormonal contraceptive users and non-users respectively.

There was a 10.14 mg/dl increment of mean serum TG level among hormonal contraceptive users (134.64 ± 18.31 mg/dl) than non-users (124.5 ± 18.38 mg/dl), and the difference was statistically significant (p = 0.000). The maximum serum TG level in hormonal contraceptive users was 171.8 mg/dl and 158 mg/dl in non-users group. While, the minimum serum TG in hormonal contraceptive users was 96.8 mg/dl and 91.2 mg/dl in non-users. Twenty three (24.7%) of hormonal contraceptive users and 9 (9.6%) non-users’ women showed with TG value ≥ 150 mg/dl (Table 2).

**Table 2:** Mean serum level of TC, TG, HDL-c and LDL-c among study participants in Goba Town Public Health facilities, Bale, Southeast Ethiopia, 2022

Lipid profile	Hormonal contraceptives		Mean difference	P-value
	User (n = 93)	Non-users (n = 93)		
TC	151.00 ± 22.67	137.48 ± 22.65	+ 13.52	0.000*
TG	134.64 ± 18.31	124.5 ± 18.38	+ 10.14	0.000*
LDL-c	67.52 ± 10.11	58.96 ± 12.12	+ 8.56	0.000*
HDL-c	59.34 ± 7.83	59.86 ± 6.68	− 0.52	0.629

Where: (−) = Decreased from controls, (+) = Increased from controls, values are represented as Mean ± Standard Deviation (mg/dl), * = statistically significant, p-values were obtained by student’s independent t-test. TC: Total cholesterol, TG: Triglyceride, LDL-c: Low density lipoprotein cholesterol, HDL-c: High density lipoprotein cholesterol

Results of our study indicated that, mean serum LDL-c levels in hormonal contraceptive users (67.52 ± 10.11 mg/dl) was significantly higher (p = 0.000) by 8.56 mg/dl than that of non-users (58.96 ± 12.12 mg/dl). The maximum serum LDL-c level among hormonal contraceptive users was 91.6 mg/dl and non-user was 89 mg/dl. Whereas, the minimum serum LDL-c among hormonal contraceptive user was 41.2 mg/dl and non-user was 32.4 mg/dl.

Serum HDL-c level in hormonal contraceptive users was decreased by 0.52 mg/dl in comparison with non-users. Mean serum HDL-c level in hormonal contraceptive users was 59.34 ± 7.83 mg/dl and 59.86 ± 6.68 mg/dl in non-users, but the difference was not statistically significant (p = 0.629, Table 2), the range being 42–74.1 mg/dl and 44–76.1 mg/dl in hormonal contraceptive users and control group, respectively. There was no reported result with HDL-c value < 40 mg/dl in both groups (Table 3).

**Table 3:** Minimum and maximum mean values of lipid profiles among hormonal contraceptives user and non-user in Goba Town Public Health facilities, Bale, Southeast Ethiopia, 2022

Lipid profile	Hormonal contraceptives	
	User (n = 93)	Non-User (n = 93)
TC (in mg/dl)		
Minimum	102.6	100.2
Maximum	196.7	180.2
TG (in mg/dl)		
Minimum	96.8	91.2
Maximum	171.8	158
LDL-c (in mg/dl)		
Minimum	41.2	32.4
Maximum	91.6	89
HDL-c (in mg/dl)		
Minimum	42	44
Maximum	74.1	76.1

TC: Total cholesterol, TG: Triglyceride, LDL-c: Low density lipoprotein cholesterol, HDL-c: High density lipoprotein cholesterol

### Lipid profile status among hormonal contraceptive users

Our study showed that, there was a significant increase in TC among women using DMPA (169.51 ± 15.82 mg/dl) than POP (145.75 ± 7.50 mg/dl) and implanon (133.54 ± 21.15) (p = 0.000). There was also a significant difference (p = 0.000) between three groups hormonal contraceptive users. The mean difference between DMPA and POP users were 23.76 mg/dl; POP and implanon users were 12.21 mg/dl; and DMPA and implanon users were 35.97 mg/dl.

The mean serum TG levels among DMPA users was 169.51 ± 15.82, POP users 145.75 ± 7.50 and implanon users 133.54 ± 21.15. There was a significant difference (p = 0.000*) in mean serum level of TG between three groups hormonal contraceptive users.

There was a 9.07 mg/dl increment of serum LDL-c level in DMPA users (75.58 ± 7.21 mg/dl) and 7.55 mg/dl increment of serum LDL-c level in POP users (66.51 ± 6.04 mg/dl) when compared to implanon users (58.96 ± 7.87 mg/dl) (p = 0.000). The mean difference between DMPA and implanon users was 16.62 mg/dl. The mean difference between the three groups of HC users were statistically significant (p = 0.000).

The mean serum HDL-c level in DMPA users and POP was decreased by 18.45 mg/dl and 10.01 mg/dl respectively when compared with implanon users. Mean serum HDL-c level of implanon users (66.48 ± 3.65) was significantly higher than that of DMPA (48.03 ± 7.06) and POP (56.47 ± 6.34) mg/dl users (p = 0.000, Table 4).

**Table 4:** Mean serum level of lipid profile among hormonal contraceptives user in Goba Town Public Health facilities, Bale, Southeast Ethiopia, 2022

Lipid profile	Hormonal contraceptive user			P-value
	DMPA (n = 37)	POP (n = 24)	Implanon (n = 32)	
TC	169.51 ± 15.82	145.75 ± 7.50	133.54 ± 21.15	0.000*
TG	141.69 ± 19.15	138.69 ± 7.21	123.45 ± 18.13	0.000*
LDL-c	75.58 ± 7.21	66.51 ± 6.04	58.96 ± 7.87	0.000*
HDL-c	48.03 ± 7.06	56.47 ± 6.34	66.48 ± 3.65	0.000*

Values are represented as M ± SD (mg/dl), * = statistically significant, p-values were obtained by One-way ANOVA. DMPA: Depo-Medroxyprogesterone Acetate, POP: Progesterone only pills

### Lipid profile in relation to the duration of hormonal contraceptive use

**Fig. 1:**
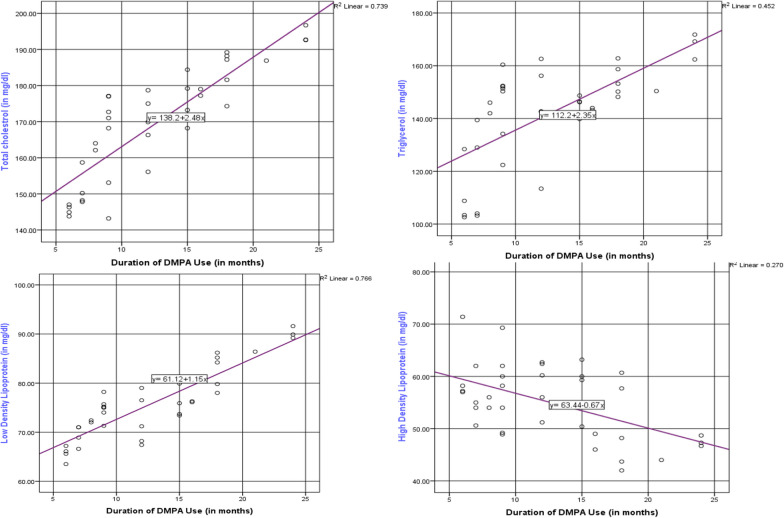
The correlation between lipid profiles and duration of DMPA use in Goba Town Public Health Facilities, Bale, Southeast Ethiopia, 2022

As the duration DMPA use increases by 1 month, the serum total cholesterol level of women increases by 2.48 mg/dl. There was significant (p = 0.000), strong and positive (r = 0.86) relationship between the duration of DMPA use and total cholesterol***.*** As the duration DMPA use increases by 1 month, the serum triglycerol level of women increases by 2.35 mg/dl. There was significant (p = 0.000), moderate and positive (r = 0.67) relationship between the duration of DMPA use and triglycerol***.*** As the duration DMPA use increases by 1 month, the serum LDL-c level of women increases by 1.15 mg/dl. There was significant (p = 0.000), strong and positive (r = 0.87) relationship between the duration of DMPA use and LDL-c. As the duration DMPA use increases by 1 month, the serum HDL-cl level of women decreases by 0.67 mg/dl. There was significant (p = 0.000), moderate and negative (r = − 0.51) relationship between the duration of DMPA use and LDL-c (Fig. 1).

**Fig. 2:**
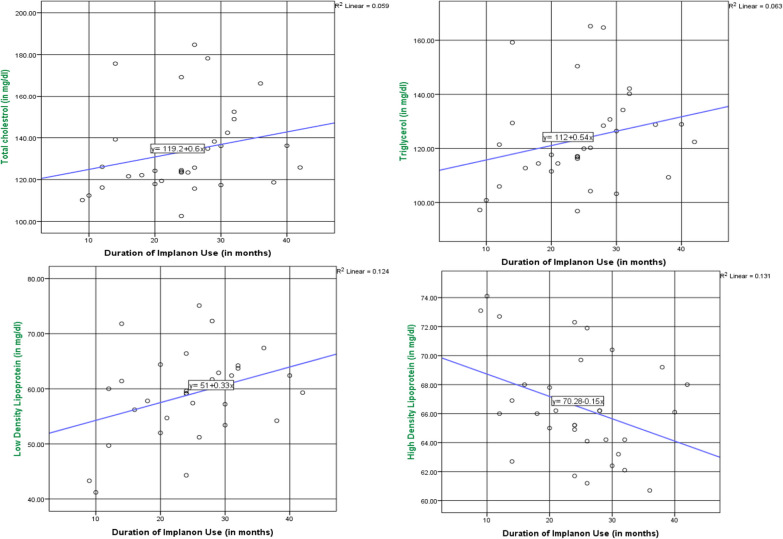
The correlation between lipid profiles and duration of Implanon use in Goba Town Public Health Facilities, Bale, Southeast Ethiopia, 2022

As the duration implanon use increases by 1 month, the serum total cholesterol level of women increases by 0.6 mg/dl. There was non-significant, weak and positive (r = 0.24) relationship between the duration of implanon use and total cholesterol***.*** As the duration implanon use increases by 1 month, the serum triglycerol level of women increases by 0.54 mg/dl. There was non-significant, weak and positive (r = 0.25) relationship between the duration of implanon use and triglycerol. As the duration implanon use increases by 1 month, the serum LDL-c level of women increases by 0.33 mg/dl. There was significant (p = 0.045), weak and positive (r = 0.35) relationship between the duration of implanon use and total LDL-c. As the duration implanon use increases by 1 month, the serum HDL-c level of women decreases by 0.15 mg/dl. There was non-significant, weak and negative (r = − 0.36) relationship between the duration of implanon use and HDL-c (Fig. 2).

**Fig. 3:**
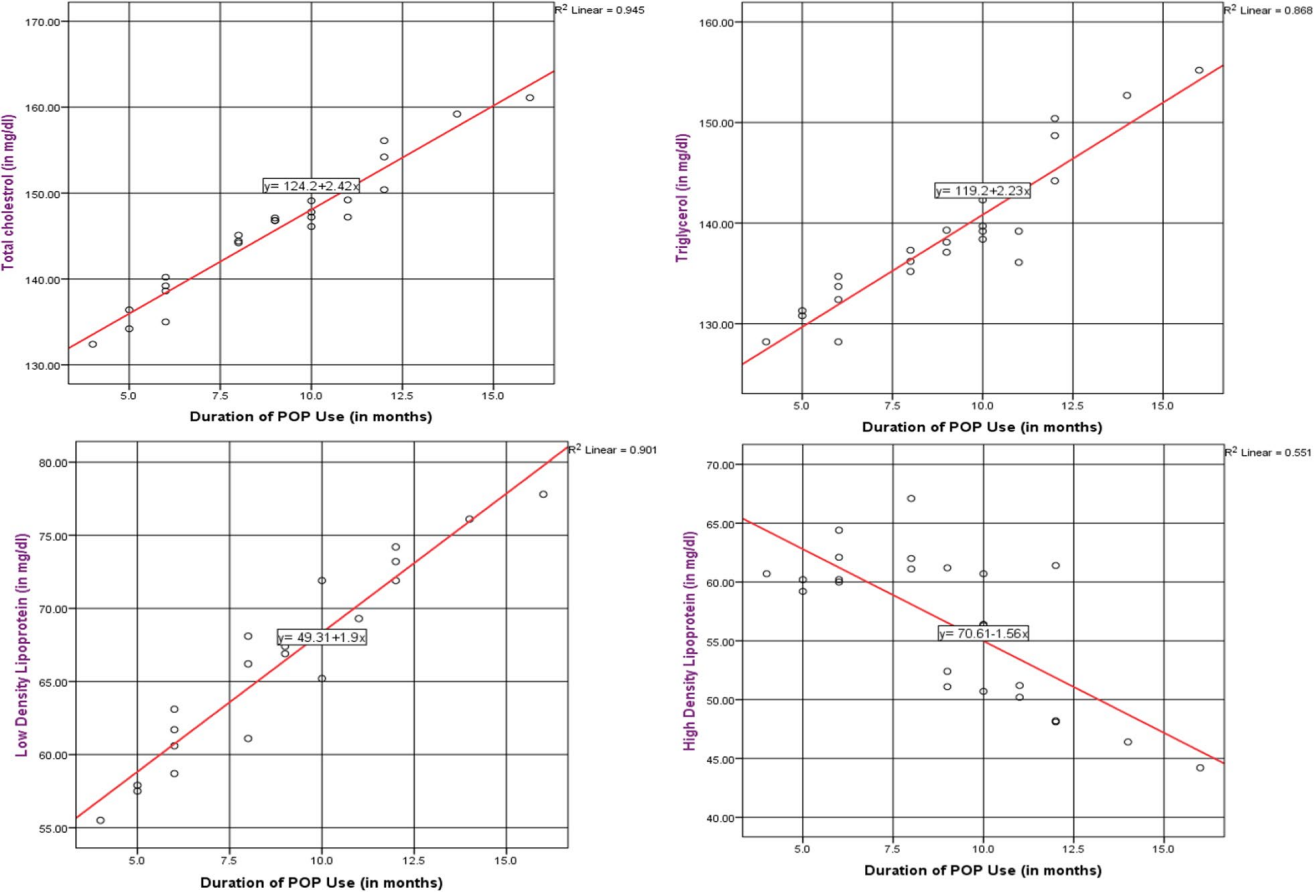
The correlation between lipid profiles and duration of POP use in Goba Town Public Health Facilities, Bale, Southeast Ethiopia, 2022

As the duration POP use increases by 1 month, the serum total cholesterol level of women increases by 2.42 mg/dl. There was significant (p = 0.000), strong and positive (r = 0.97) relationship between the duration of POP use and total cholesterol. As the duration POP use increases by 1month, the serum triglycerol level of women increases by 2.23 mg/dl. There was significant (p = 0.000), strong and positive (r = 0.93) relationship between the duration of POP use and triglycerol. As the duration POP use increases by 1 month, the serum LDL-c level of women increases by 1.9 mg/dl. There was significant (p = 0.000), strong and positive (r = 0.94) relationship between the duration of POP use and LDL-c. As the duration POP use increases by 1 month, the serum HDL-c level of women decreases by 1.56 mg/dl. There was significant (p = 0.000), moderate and negative (r = − 0.74) relationship between the duration of POP use and HDL-c (Fig. 3).

### Castelli Risk Index

There was a significant (p = 0.001) difference in Castelli Risk Index I and II between hormonal contraceptive users and non-users. It means that, the use of hormonal contraceptives was associated with the development of cardiovascular disease such as coronary heart disease, congestive heart failure and blood pressure. Castelli Risk Index I (TC/HDL-c) was high risk. Whereas, Castelli Risk Index II (LDL-c/HDL-c) was an intermediate risk factors for the development of cardiovascular disease in hormonal contraceptive users (Table 5).

**Table 5:** Castelli Risk Index I and II of hormonal contraceptive users and non-users in Goba Town Public Health Facilities, Bale, Southeast Ethiopia, 2022

Castelli Risk Index	HC users	Non-users	Mean difference	p-value
TC/HDL-c	2.63 ± 0.69	2.32 ± 0.47	0.31	0.001
LDL/HDL-c	1.18 ± 0.32	0.99 ± 0.25	0.19	0.001

Values are represented as M ± SD (mg/dl), * = statistically significant, P-values were obtained by student’s independent t-test, Casteli risk index: < 0.11 (low risk), 0.11–0.21 (intermediate risk), > 0.21 (high risk)

## Discussions

The findings of this study indicated that both groups of hormonal contraceptive users and non-users had similar age, ethnicity, religion, marital status, educational level, occupation, monthly income, and place of living. This demonstrates that the differences in these two groups' lipid profile values were not caused by socio-demographic characteristics. The results of this study are consistent with those of other studies [8, 17].

The mean serum TC level of hormonal contraceptive users was significantly increased by 13.52 mg/dl in comparison with non-users’ groups. This finding is consistent with the previous study done in Pakistan [11], Ghana [8], Nepalese [22] and Egypt [23]. The majority of currently used hormonal contraceptives are progesterone-only contraceptives, which may be the cause of the rise in TC among hormonal contraceptive users [2]. Thus, women using hormonal contraceptives have elevated synthetic progestin and low circulating estrogen [17]. Studies revealed a non-significant difference in the mean serum TC level between the two groups [16–18]. The probable reasons for the discrepancy among the studies might be due to the difference in age of study participants, sample size and study design.

Compared to POP and implanon users, women who use DMPA have a considerably greater rate of TC. The mean difference between users of DMPA and POP was 23.76 mg/dl, between users of POP and implanon was 12.21 mg/dl, and between users of DMPA and implanon was 23.76 mg/dl. This result is consistent with a prior study conducted in Ghana that found a substantial difference in the mean serum TC levels of DMPA, Implant, and POP [24]. They claimed that DMPA users had higher mean serum TC levels than POP and implanon users. The current finding is also consistent with the research done by Yadav et al. reported that Implanon users had a lower mean serum TC level than DMPA and POP users [22]. Our study's findings are comparable to those of a study carried out in Benin by Aisien and Idogun [25]. DMPA is one of the most common progesterone only contraceptive with 150 mg/ml of progestin’s composition. Progesterone only pills is composed 75 mg of progestin; whereas, implanon rod consists of an ethylene vinyl acetate copolymer core, containing 68 mg of the synthetic progestin with the release rate of 60–70 μg/day in week 56 to 25–30 μg/day at the end of the third year [17, 26, 27]. Thus, the variation in progestin composition in different hormonal contraceptives may affect lipid profile differently.

In this study, there was a 10.14 mg/dl increment of serum TG level in hormonal contraceptive users when compared than non-users. This result is in line with findings from other studies [28–30]. Low estrogen and high progesterone concentration that inhibit the serum HDL-c synthesis may contribute for elevation of TG in hormonal contraceptive users. In contrast, other studies reported a decrease in serum triglyceride in hormonal contraceptive users among hormonal contraceptive users. The discrepancy may be as a result of age of study participants, food habits and life style [8, 31].

Users of DMPA had mean blood TG levels of 169.51 ± 15.82; POP users, 145.75 ± 7.50; and implanon users, 133.54 ± 21.15. This showed that the three groups of hormonal contraception users had significantly different mean blood levels of TG. This finding is in line with the previous study done in Egypt reported DMPA users had a higher mean serum TG level than implanon users [23]. The current finding is also consistent with a study done in Nepal that found POP had higher mean serum TG levels than implanon users [22]; and in Pakistan, the serum TG level of implanon users was lower than that of DMPA and POP users [30]. The progestin content of DMPA, POP, and implanon may differ, which could have an impact on how the serum TG level is affected. This study differs from those carried out Ghana [24] and Nigeria [26, 29]. These discrepancies might be due to research design and participant genetic variability.

Serum LDL-c levels in hormonal contraceptive users was significantly higher by 8.56 mg/dl than that of non-users. This is consistent with studies done in Egypt [18], Nepal [22], Thailand [32] and Iraq [28]. The fact that estrogen is known to improve lipid profiles could be the cause of this rise in LDL-c in hormonal contraception users and it have anti-atherosclerotic effects through accelerated liver cholesterol conversion to bile acids and increased expression of LDL-C receptors on cell surfaces, which results in augmented clearance of LDL-c from the plasma [33]. Since women using HC have low circulating estrogen, it results in an elevation of serum LDL-c concentration. However, some studies reported that hormonal contraceptive use did not cause significant change in mean serum LDL-c level [16, 17]. Jamil and Siddiq [12]; and Lizarelli et al. [21] reported hormonal contraceptive users group has had lower values of LDL-c than other non-hormonal contraceptive users. Different eligibility criteria and life styles might be contributed for this discrepancy.

There was a 9.07 mg/dl increment of serum LDL-c level in DMPA users and 7.55 mg/dl increment of serum LDL-c level in POP users when compared to implanon users. The mean difference between DMPA and implanon users was 16.62 mg/dl. This finding is in line with the previous study done in Nepal [22], Ghana [24], and Iraq [25]. High progestin concentration in DMPA (150 mg) in comparison with POP (75 mg) and implanon (68 mg) and: low estrogen concentration can affect serum lipid profile differently among different hormonal contraceptive users. In contrast to present study, a study conducted by Okeke et al., reported that LDL-c fraction was significantly reduced in women on oral and injectable contraceptives [29]; a decrease in LDL-c in DMPA users [12]. This discrepancy could be due to the difference in study setting, differences in lifestyle, including diet, alcohol intake habit, and physical activity.

Serum HDL-c level in hormonal contraceptive users was decreased by 0.52 mg/dl from non-users. This result was in line with other studies [8, 29, 30, 34].

The serum HDL-c level in DMPA and POP users was decreased by 18.45 mg/dl and 10.01 mg/dl respectively when compared with implanon users; and the mean serum HDL-c level of implanon users was significantly higher than that of DMPA and POP users. The possible explanation might be progestin concentration of hormonal contraceptives and serum HDL-c was inversely proportional to each other. The progestin release rate of implanon is also decline over. Since the progestin concentration of implanon is lower than that of DMPA and POP, the serum HDL-c level of implanon users were higher than DMPA and POP users. This findings are in agreement with other studies [17, 18, 21].

In accordance with our research, the blood levels of TC, TG, and LDL-c greatly rise as the duration of DMPA and POP use increases, while the serum level of HDL-c dramatically declines. The duration of implanon use had no effect on the serum levels of TC, TG, or HDL-c; nevertheless, the serum level of LDL-c dramatically rises with increasing implanon usage time. Similar findings were supported increase in serum level of LDL-c among implanon users [8, 24, 35]. As the duration of hormonal contraceptive increases, the deposition of progestin in the tissue increases which alter lipid metabolism, reduce endothelial macrophages that have been taken up TG and LDL-c aggregates, inhibit HDL-c synthesis which prevents LDL-c aggregation [30]. A study conducted by Al-youzbaki et al. [16], and Fikadie [36] reported non-significant relationship between the duration of DMPA use and lipid profile. Age of study participants, eligibility criteria, length of use of HC and study setting could be contributing to the discrepancy of present and prior result.

The ratio of TC to HDL-c and LDL-c to HDL-c were significantly increases in hormonal contraceptive users in comparison with non-users. The possible physiologic explanation for difference might be lipid peroxide product malondialdehyde was suppressed by hormonal contraceptive result in elevated blood lipid levels are risk factors for atherosclerosis and coronary heart disease [16].

The present study was similar with the previous finding of Dilshad et al., and Bawah et al., there was a significant difference in TC/HDL-c and LDL-c/HDL-c ratio between HC users and non-users. Another study done in Nigeria by Mbakwem et al., also reported a significant difference between two groups. Our finding was also in line with study conducted by Baghamad et al., reported a significant difference in Castelli risk index I and II among HC users and non-users [33]. However, our result was in contrast with a previous study conducted by previous studies [8, 17, 29]. Variations in findings could also be due to differences in study settings and food habits. Most Ethiopians usually utilize imported edible palm oil containing a large proportion of saturated fatty acid that raises TC/HDL-c and LDL-c to HDL ratio.

## Conclusions

Users of hormonal contraceptives had higher mean serum levels of TC, TG, and LDL. HDL levels in the serum, however, were not significantly changed. The mean serum level of lipid profiles were different between DMPA, Implanon and POP users. DMPA user had highest mean serum level of TC, TG and LDL-c, but had lowest mean serum level of HDL-c in comparison with POP and implanon users. There is no relationship between the serum level of TC, TG, HDL-c; and duration of implanon use. But, the serum level of LDL-c was significantly increases as the duration of implanon use increases. There was also a significant difference in Castelli Risk Index I and II between hormonal contraceptive users and non-users.

Hormonal contraceptive users recommended regular evaluation of lipid profile at each visit of family planning unit. Routine evaluation of lipid profiles is advisable before initiating hormonal contraceptive. Women who had cardiovascular problem (myocardial infarction, congestive heart failure, increased blood pressure, pulmonary embolism, tachycardia and thromboembolic disorders) should not be recommended for using DMPA and POP as a contraceptive method. If the use hormonal contraceptive was mandatory, implanon is more preferable than DMPA and POP.

Hormonal contraceptive users were recommended to use other options of contraceptives (non-hormonal contraception). This study is cross sectional in design, further longitudinal study (cohort in design) with larger sample size is need to be conducted to evaluate whether the changes in lipid profile is associated with use of hormonal contraceptive.

## Data Availability

The datasets used to support this paper's conclusions are presented in the article.

## Abbreviations

DMPA: Depo-Medroxyprogesterone Acetate
HC: Hormonal contraceptive
HDL-c: High density lipoprotein cholesterol
LDL-c: Low density lipoprotein cholesterol
NO: Nitric oxide
POP: Progesterone only pills
TC: Total cholesterol
TG: Triglyceride
v-LDL-c: Very low density lipoprotein cholesterol
